# Kinematic analysis of social interactions deconstructs the evolved loss of schooling behavior in cavefish

**DOI:** 10.1371/journal.pone.0265894

**Published:** 2022-04-06

**Authors:** Adam Patch, Alexandra Paz, Karla J. Holt, Erik R. Duboué, Alex C. Keene, Johanna E. Kowalko, Yaouen Fily

**Affiliations:** 1 Wilkes Honors College, Florida Atlantic University, Jupiter, FL, United States of America; 2 Jupiter Life Science Initiative, Florida Atlantic University, Jupiter, FL, United States of America; 3 Department of Biological Sciences, Florida Atlantic University, Jupiter, FL, United States of America; 4 Department of Biological Sciences, Lehigh University, Bethlehem, PA, United States of America; 5 Department of Biology, Texas A&M University, College Station, TX, United States of America; Centre National de la Recherche Scientifique, FRANCE

## Abstract

Fish display a remarkable diversity of social behaviors, both within and between species. While social behaviors are likely critical for survival, surprisingly little is known about how they evolve in response to changing environmental pressures. With its highly social surface form and multiple populations of a largely asocial, blind, cave-dwelling form, the Mexican tetra, *Astyanax mexicanus*, provides a powerful model to study the evolution of social behavior. Here we use motion tracking and analysis of swimming kinematics to quantify social swimming in four *Astyanax mexicanus* populations. In the light, surface fish school, maintaining both close proximity and alignment with each other. In the dark, surface fish no longer form coherent schools, however, they still show evidence of an attempt to align and maintain proximity when they find themselves near another fish. In contrast, cavefish from three independently-evolved populations (Pachón, Molino, Tinaja) show little preference for proximity or alignment, instead exhibiting behaviors that suggest active avoidance of each other. Two of the three cave populations we studied also slow down when more fish are present in the tank, a behavior which is not observed in surface fish in light or the dark, suggesting divergent responses to conspecifics. Using data-driven computer simulations, we show that the observed reduction in swimming speed is sufficient to alter the way fish explore their environment: it can increase time spent exploring away from the walls. Thus, the absence of schooling in cavefish is not merely a consequence of their inability to see, but may rather be a genuine behavioral adaptation that impacts the way they explore their environment.

## Introduction

Social behaviors differ dramatically across species and correspond to the animals’ unique evolutionary histories and ecological factors including predation threat, foraging strategy, sexual selection pressure, and available sensory inputs [1–4]. Comparisons between closely related species or populations within the same species provide insight into the variability of social behavior, its dependence on ecological factors, and its genetic and neural bases [5].

Shoaling, during which fish maintain proximity but are not aligned with one another, and schooling, in which fish swim together in aligned groups, are behaviors that can vary dramatically between populations of the same species and are influenced by both genetic and environmental factors [6–10]. For example, in threespine sticklebacks, natural polymorphisms in the *ectodysplasin a (eda)* locus contribute to the differences in inter-fish alignment observed between benthic and marine populations [10–13]. In Trinidadian guppies, individuals descended from low-predation environments display diminished social cohesions [8, 9, 14–18], though exposure to alarm pheromones leads to the formation of tighter schoolss [15]. Sensory cues also influence social cohesion, such as in sticklebacks, in which modulation of visibility (light vs dark) and acoustic noise leads to differences in group dynamics [19]. Together, these findings illustrate that while genetic differences contribute to natural variation in schooling and shoaling behaviors, these social traits also demonstrate plasticity and are amenable to being shaped by environmental cues.

Cave organisms, including bats [20], crayfish [21], cardinalfish [22], Atlantic mollys [23–25], and the Mexican tetra [26] have proven useful for investigating the intersection of environment and social dynamics. The Mexican tetra, *Astyanax mexicanus*, is exceptionally well-suited for this purpose because it exists in two forms that inhabit locations that differ in parameters known to influence social behavior, including predation intensity, salience of sensory cues, and nutrient availability [27]. Surface forms of *A. mexicanus* inhabit above-ground rivers and streams through Mexico and southern Texas, and are highly social. By contrast, there are at least 30 populations of cave-dwelling fish found in the Sierra de El Abra and Sierra de Guatemala regions of Northeast Mexico, which have been shown to lack social cohesion with one another [28–31]. This cave form evolved multiple times independently in different caves [32]. Importantly, the differences in social behavior between surface and cave forms have been observed both in the field and in the lab [33]. The loss of schooling is just one in a series of morphological and behavioral differences cavefish have evolved, which include eye loss, increased sensitivity of the mechanoreceptive lateral line, and a reduction in aggression [7, 34–37]. The influence of eye-loss on school behavior in A. mexicanus has been a source of debate. Previous data have revealed that vision-deprived surface fish do not school, suggesting the lack of visual inputs may explain the lack of schooling in cavefish. However, some surface-cave hybrids that respond to visual cues do not school either, suggesting the loss of schooling may also have a vision-independent component [7]. Modern advances in computer vision and modeling of collective behavior provide an unprecedented opportunity to resolve the role of eye loss on schooling behavior. Motion tracking and kinematic analysis have been used to probe schooling interactions in several fish species [14, 16, 38–42]. Pair correlation functions (probability distributions involving the position and orientation of one fish relative to another) are particularly useful to disentangle positional and orientational interactions and build mechanistic models of collective motion that can simulate the trajectories of a group of virtual fish [38–41].

In this paper, we use a motion-tracking-based schooling assay to address four questions related to schooling and its evolution in *A. mexicanus*: (i) What is an appropriate set of metrics to quantify social swimming in *A. mexicanus* beyond schooling/not schooling? (ii) Can those metrics clarify the connection between the loss of schooling and the loss of vision? (iii) Can those metrics identify differences between different cave populations? (iv) What fundamental behavioral components should a future mechanistic model of *A. mexicanus* swimming include?

## Results

### Boundary effects and the importance of tank design

Surface and cave populations of *A. mexicanus* have been studied extensively for evolved differences in behaviors including locomotion, schooling, and sleep. While these studies reveal critical differences in behavior between the two forms, they use assays which do not always reflect motion under natural conditions including fish interacting through a transparent partition, fish swimming with a school of artificial fish, and small rectangular tanks that bias the fish’s orientation and turning behavior [7, 10, 43–46]. Unbiased turning statistics are particularly important for developing mechanistic schooling models, which normally start by modeling unprompted changes of speed or orientation, i.e., changes that are not triggered by the proximity of a wall or other fish, before modeling the effect of the walls (by comparing trajectories near/far from the wall) and the other fish (by comparing trajectories near/far from other fish). With this in mind, we first examine the swimming dynamics of single fish.

**Fig 1:**
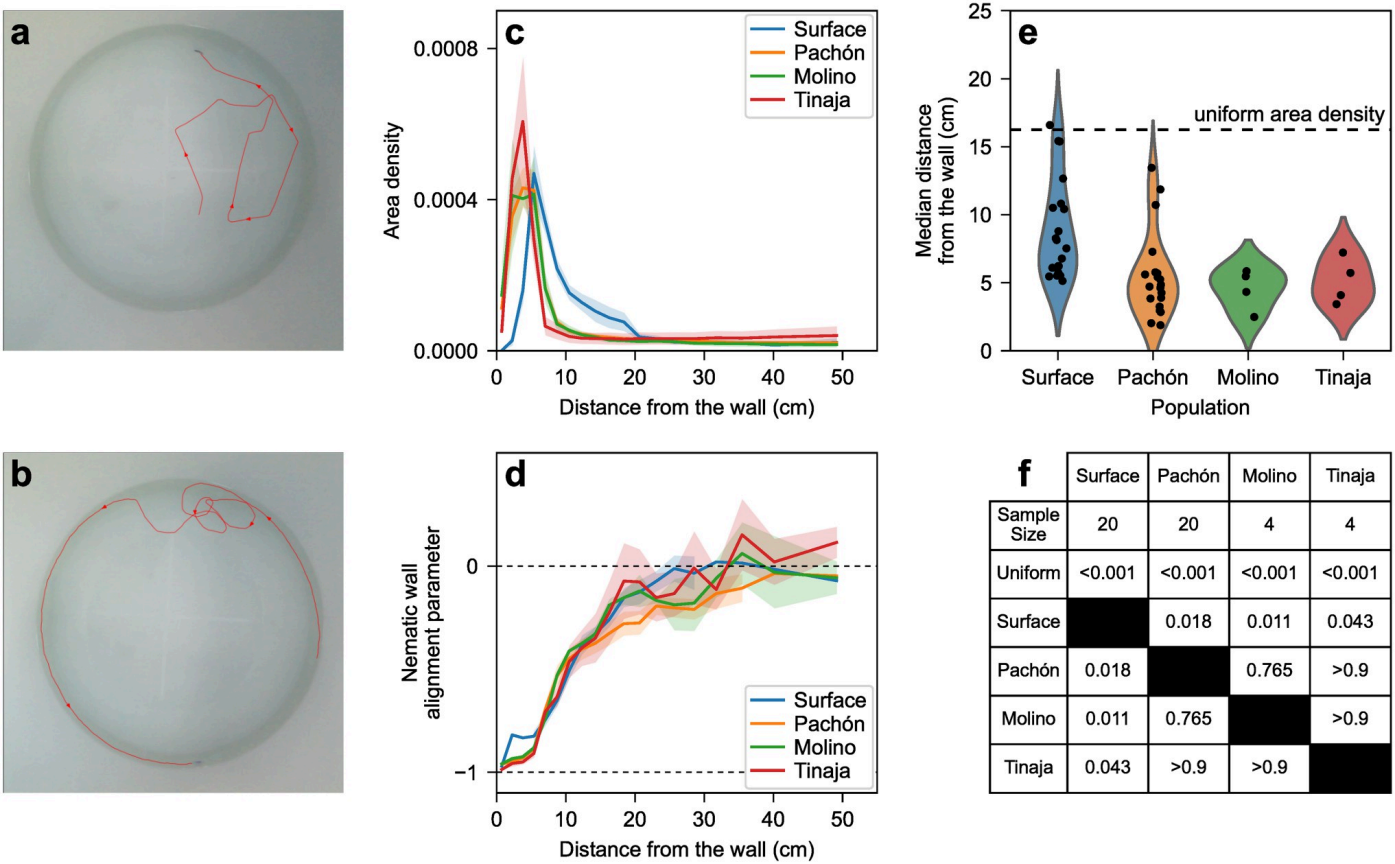
(a) Snapshot from a video of a single surface fish. The red line is the trajectory over the 25 seconds leading to the snapshot. (b) Similar snapshot and 25 s-trajectory from a video of a single Pachón cavefish. (c) Fish density probability per unit area as a function of the distance from the wall for single fish trials for each population. The distance varies between 0 cm (right against the wall) and 55.5 cm (center of the tank). The halo around each curve corresponds to one standard error on either side of the mean. (d) Average nematic alignment parameter relative to the wall (cos(2*θ*) where *θ* is the angle between the fish’s heading and the closest wall’s normal vector) as a function of the distance to the wall. Values near -1 indicate strong alignment parallel to the wall. Values near 0 suggest no favored orientation. The halo around each curve corresponds to one standard error on either side of the mean. (e) Median distance from the wall in each trial, by population. The dashed line is the median distance with a uniform area density (tank radius times $\left(1-1/\sqrt{2}\right)$; half of the tank’s area is within that distance of the wall). (f) Median distance from the wall statistics. Every population is different from the uniform case (Shapiro test, then T-test if normal or Wilcoxon test if not, *N*_Su_ = 20, *N*_Pa_ = 20, *N*_Mo_ = 4, *N*_Ti_ = 4, *p* < 0.001 for every population). Cave populations are different from surface fish, but not from each other (Kruskal-Wallis test, *p* = 0.0015, then Games-Howell test, *p*_Su−Pa_ = 0.018, *p*_Su−Mo_ = 0.011, *p*_Su−Ti_ = 0.043, *p*_Pa−Mo_ = 0.765, *p*_Pa−Ti_ > 0.9, *p*_Mo−Ti_ > 0.9).

Individual fish from surface, Pachón, Tinaja, or Molino populations were filmed in a circular tank whose diameter was 20 times the fish’s body lengths (Fig 1a and 1b). The circular shape was picked because it does not favor any overall orientation. The tank was designed to be shallow so motion was mostly two-dimensional and could be filmed from above with minimal information loss. Fish were filmed for 20 minutes following a 10-minute acclimation period.

To assess how far the effect of the walls influences the dynamic movements of the fish, we measured their distance to and orientation with respect to the nearest wall in lighted conditions (Fig 1c and 1d). Fish from both surface and cave populations spent the vast majority of their time near the wall (Fig 1c). However, cavefish stay closer to the wall than surface fish, possibly representing thigmotaxis [47]. The fish density per unit area (probability of finding a fish per unit area of the tank across all frames) as a function of the distance to the wall reveals that cavefish mostly stay between 2–5 cm, while surface fish have a broader peak at 7 cm. This is also reflected in the median distance from the wall, which is biased toward the wall for both surface and cave populations, suggesting proximity to a wall is a conserved trait. However, while no significant differences in the median distance from the wall were observed between the different cave populations, detailed analysis revealed that cave populations stayed closer to the wall relative to surface animals (Fig 1e). For every population, the density per unit area plateaus between 10 and 20 cm from the wall, suggesting that at 10–20 cm the location of a fish no longer depends on the distance to the wall (Fig 1c). To further assess how proximity to the wall influences swimming behavior, we assessed the nematic wall alignment, a metric that measures the orientation of a fish relative to the nearest wall. A value of 0 suggests the fish orients independently of the wall, deviations toward 1 indicate the fish is more likely to be perpendicular to the wall, and deviations toward −1 indicate the fish is more likely to be parallel to the wall.

Further, while fish near the wall display nematic wall-alignment parameter values near -1, demonstrating that they stay mostly parallel to the wall, fish located more than 20 cm from the wall exhibit values near 0, suggesting they orient independently of the wall (Fig 1d).

Overall, this shows that the effect of the walls on fish behavior remains strong up to at least 20 cm from the wall. Thus, when designing a tank to quantify the dynamics of surface and cave *A. mexicanus* in situations where they are not dominated by the wall, one should therefore use a tank larger than 40 cm in diameter. Additionally, some results from the literature may have to be reinterpreted. For example, Sharma et al. found that surface fish occupy the entirety of a 30 cm-diameter tank [47] whereas cavefish stay near the wall. What our results suggest is that surface fish do prefer to stay near the wall, however, the entirety of a 30 cm-diameter tank is within 15 cm of the wall, which may still be near the wall for a surface fish.

### Group size affects speed regulation and the nature of turns

Schooling and shoaling are defined by the way the fish in a group position and orient themselves with respect to each other. However, the presence or absence of other fish can also affect a fish’s overall swimming behavior, regardless of its proximity to another fish. Cataloging such effects is important both for understanding the effects of the presence of conspecifics on individual fish swimming behavior and to guide future mechanistic models. Thus, we calculated the effects of group size on the fish’s speed and turning speed.

#### Isolated surface fish pause

**Fig 2:**
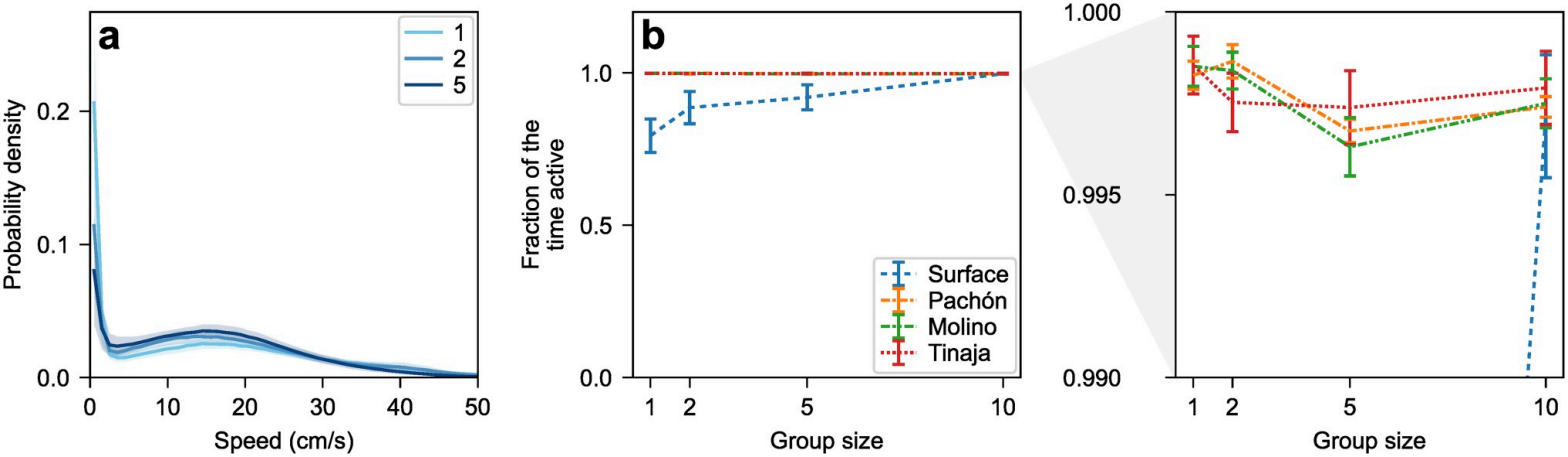
(a) Speed distribution of surface fish as a function of group size. The halo around each curve corresponds to one standard error on either side of the mean. A sharp peak near zero indicates fish are not moving. The area under the peak is the fraction of the time the fish are not moving. (b) Fraction of time spent moving faster than 1 cm/s (“active”) as a function of group size. Cavefish are almost always moving (they are active > 99% of the time in every trial regardless of population or group size), whereas surface fish are less active when they are more isolated (linear regression slope 0.02 cm/s/fish, *N* = 52, *p* = 0.007). (c) Same as (b), zooming in on the cave populations.

Surface fish, both alone and in groups, occasionally stop moving, as evidenced by the sharp zero-speed peak in their speed distributions (Fig 2a). The area under this peak represents the average fraction of the time the fish were inactive. The base of the peak is consistently located around 1 cm/s, providing a natural definition of active (moving faster than 1 cm/s) versus inactive (slower than 1 cm/s) fish. The fraction of the time surface fish were active shows a clear trend, from 73% in single-fish trials up to close to 100% in groups of 10 (Fig 2b). In contrast, cavefish keep moving regardless of the number of fish in the tank (Fig 2b). This is consistent with previous studies showing that isolated surface fish demonstrated increased stress response compared with cavefish; when surface fish are placed in a novel tank, they seek the bottom of the tank whereas cavefish do not [48].

#### Pachón and Tinaja cavefish slow down in larger groups

Given this threshold of active vs. inactive states, from this point on we focus on swimming properties in the active state, i.e., we exclude the inactive data (speed<1 cm/s) from all subsequent distributions and averages. In practice this inactivity cut only affects groups of 1, 2, and 5 surface fish, i.e., those shown in Fig 2a.

**Fig 3:**
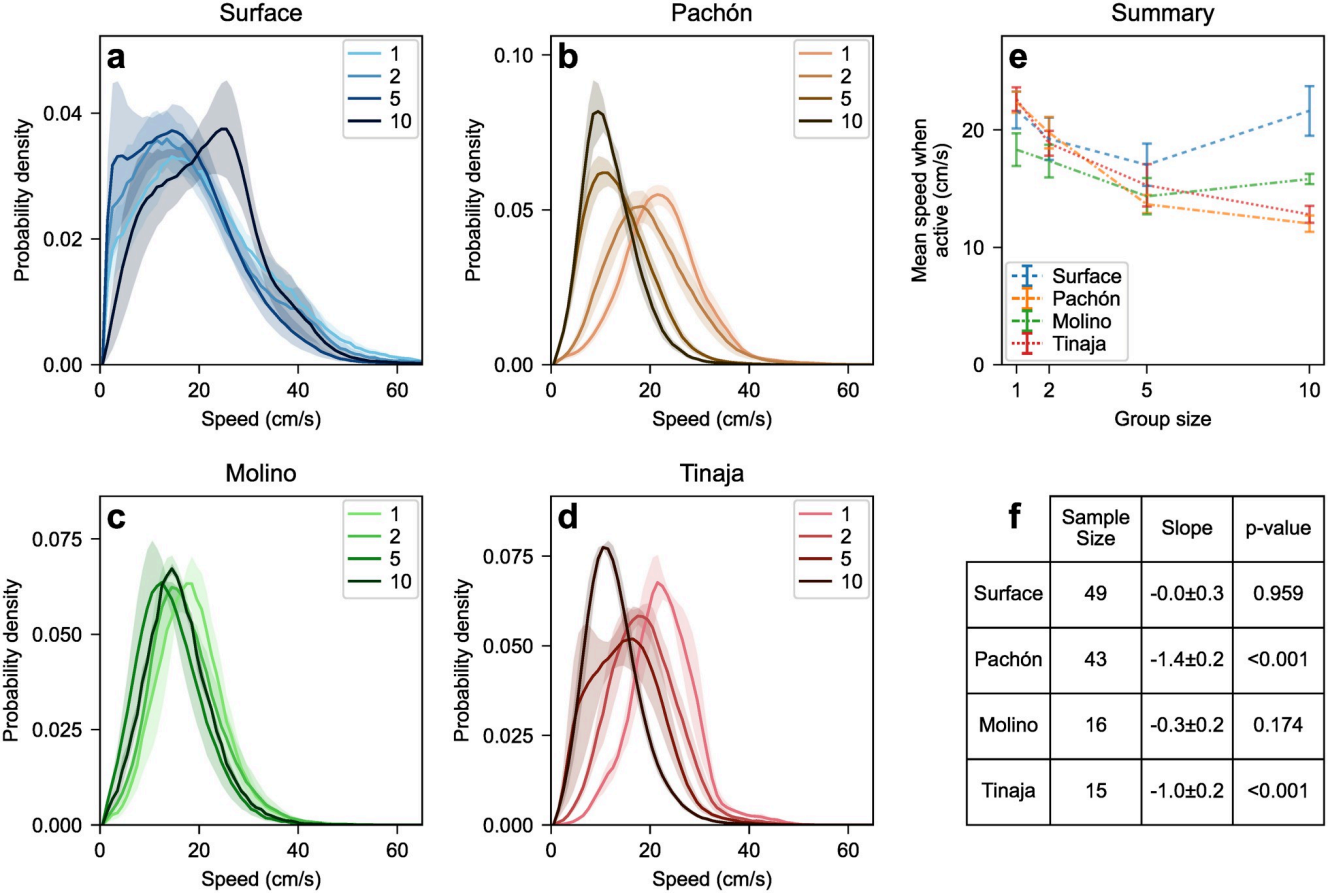
(abcd) Speed distribution as a function of group size for actively swimming (speed>1 cm/s) surface fish and three cave populations (Pachón, Molino, Tinaja). The halo around each curve corresponds to one standard error on either side of the mean. (e) Mean speed as a function of group size for all types. Pachón and Tinaja populations slow down when group size increases whereas surface and Molino show no significant trend across 1 ≤ *n* ≤ 10. (f) Mean speed linear regression slopes: *m*_Su_ = 0.0 ± 0.3 (*N* = 49, *p* = 0.959), *m*_Pa_ = −1.4 ± 0.2 (*N* = 43, *p* < 0.001), *m*_Mo_ = 0.3 ± 0.2 (*N* = 16, *p* = 0.174), *m*_Ti_ = −1.0 ± 0.2 (*N* = 15, *p* < 0.001).

Removal of inactive frames reveals that the speed distribution of surface and Molino fish depends little on the number of fish in the tank (Fig 3a and 3c). Conversely, Pachón and Tinaja cavefish noticeably slow down as the number of other fish increases (Fig 3b and 3d).

If the slowdown only occurred during encounters with other fish, we would expect the speed distribution in larger groups to consist of the same peak seen in isolated fish, plus a second peak or shoulder at smaller speeds corresponding to encounters with other fish. Similar shoulders or multiple peaks can appear when the mean speed varies from trial to trial, as is the case in larger groups of surface fish (Fig 3a). What we see instead in Pachón and Tinaja cavefish (Fig 3b and 3d) is that the entire distribution shifts to the left (to lower speeds), suggesting the slowing down is a persistent response to the presence of other fish in the tank rather than a short-lived response to being near another fish.

Remarkably, this slowdown is not limited to encounters with other fish but rather reflects the overall behavior of the fish both during and between encounters, as evidenced by the speed distribution shifting as a whole rather than developing a second, slower peak (Fig 3b). Tinaja cavefish exhibit the same slowdown with respect to group size observed in Pachón cavefish, however Molino cavefish do not (Fig 3c).

#### Surface fish turn more abruptly in smaller groups, whereas cavefish turn more abruptly in larger groups

Another fundamental quantity needed to characterize the undisturbed (by walls or other fish) motion of a fish is its distribution of turning speed (angular speed). Mechanistic models often use one of two assumptions: that the angular speed distribution follows a normal distribution, or that there is an alternation of straight bouts and instantaneous changes of direction. The former has been used to model barred flagtails [49]. The latter has been observed in rummy-nose tetra [40] and larval zebrafish [50].

**Fig 4:**
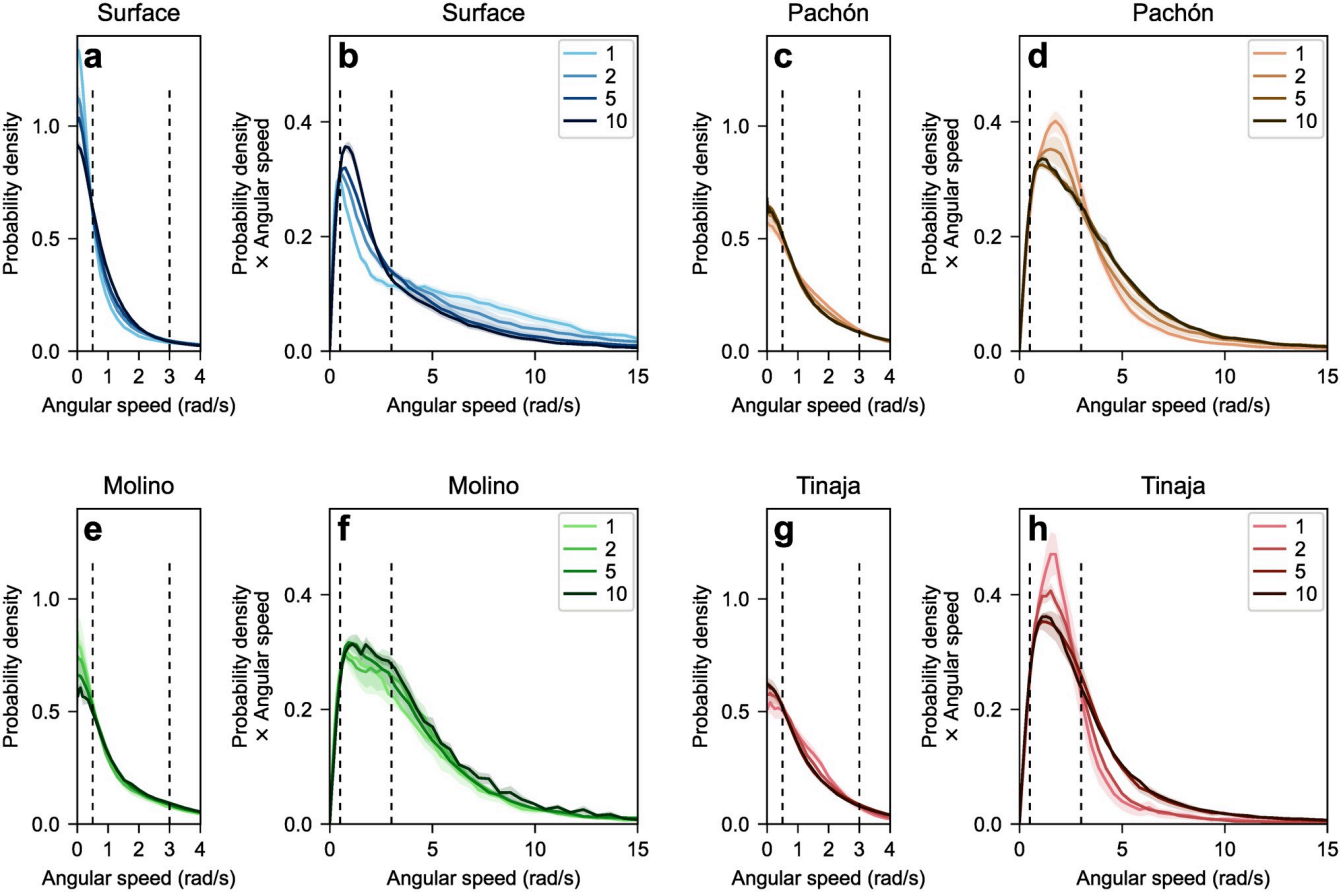
Distribution of turning speed as a function of group size for (ab) surface fish and (cdefgh) three cave populations (Pachón, Molino, Tinaja). The halo around each curve corresponds to one standard error on either side of the mean. (aceg) The left panel of each pair shows the probability density of the turning speed. It is related to the time spent turning at each turning speed. (bdfh) The right panel of each pair shows the same probability density, multiplied by the angular speed. It is related to the total angle turned at each turning speed. The two vertical dashed lines in each panel correspond to 0.5 rad/s and 3.5 rad/s, respectively.

To analyze turning speeds, we compute the probability density of turning speed (Fig 4a, 4c, 4e and 4g). Integrating the probability density between two values of the turning speed yields the fraction of the time the turning speed was between those two values. In particular, the area under the peak at zero turning speed represents the fraction of time spent going straight or nearly straight. Comparing the heights of those peaks across populations and group sizes, we find that surface fish spend more time going straight than any cave population. In surface fish and Molino fish, the height of the peak decreases with increased group size, indicating isolated fish turn less often (Fig 4a and 4e). Pachón and Tinaja fish exhibit a weaker trend in the opposite direction: isolated fish turn more often than fish in larger groups (Fig 4c and 4g). Those trends persist up to about 0.5 rad/s (first vertical dashed line in each panel of Fig 4). For reference, this is comparable to the angular speed required to follow the wall ($\frac{\text{typical}\;\text{swimming}\;\text{speed}}{\text{tank}\;\text{radius}}\approx \frac{20cm/s}{55.5cm}\approx 0.4\;\text{rad}/\text{s}$).

To assess which turning speeds contribute the most rotation, we also analyze the probability density of turning speed multiplied by the turning speed (Fig 4b, 4d, 4f and 4h). With this method, integrating between two values of the turning speed yields the total angle turned in that range of turning speed per second of the trial. Thus, the focus is on the amount of rotation done at each turning speed rather than the amount of time spent turning at each turning speed. In surface fish, increasing group size increases the amount of gradual turning (<3.5 rad/s) but decreases the amount of quick turning (>3.5 rad/s) (Fig 4b). By contrast, in Pachón and Tinaja fish, increasing group size decreases the amount of gradual turning but increases the amount of quick turning (Fig 4d and 4h). Finally, Molino fish exhibit no clear trend either way (Fig 4f).

Overall, isolated surface fish change direction more abruptly (more straight runs and more sudden turning) than isolated cavefish, whose trajectories are more rounded. This is consistent with the sample trajectories shown in Fig 4a and 4c. As group size increases, surface fish make more and rounder turns whereas Pachón fish and Tinaja fish make more sudden turns. In comparison, Molino fish turns do not change with group size.

Interestingly, both the speed analysis and the turning speed analysis suggest that Pachón and Tinaja cavefish are very similar to each other, but not as similar to Molino cavefish. Moreover, we find that Pachón and Tinaja cavefish are more different from surface fish than Molino cavefish are different from surface fish. These data are consistent with a previous study suggesting Pachon and Tinaja fish evolve from an old lineage, whereas Molino fish evolved from a newer invasion into the caves [51].

Lastly, the fact that surface fish shift from an instantaneous-turn type of dynamics when isolated to a more continuous type of turn in larger groups is significant for future mechanistic models. Those two types of turning dynamics are usually described with different types of mathematical models, therefore treating the two-fish dynamics as two single-fish dynamics plus interaction forces and torques may not be sufficient to capture the transition from isolated swimming to group swimming.

### Loss of schooling is dependent partly, but not exclusively on vision

**Fig 5:**
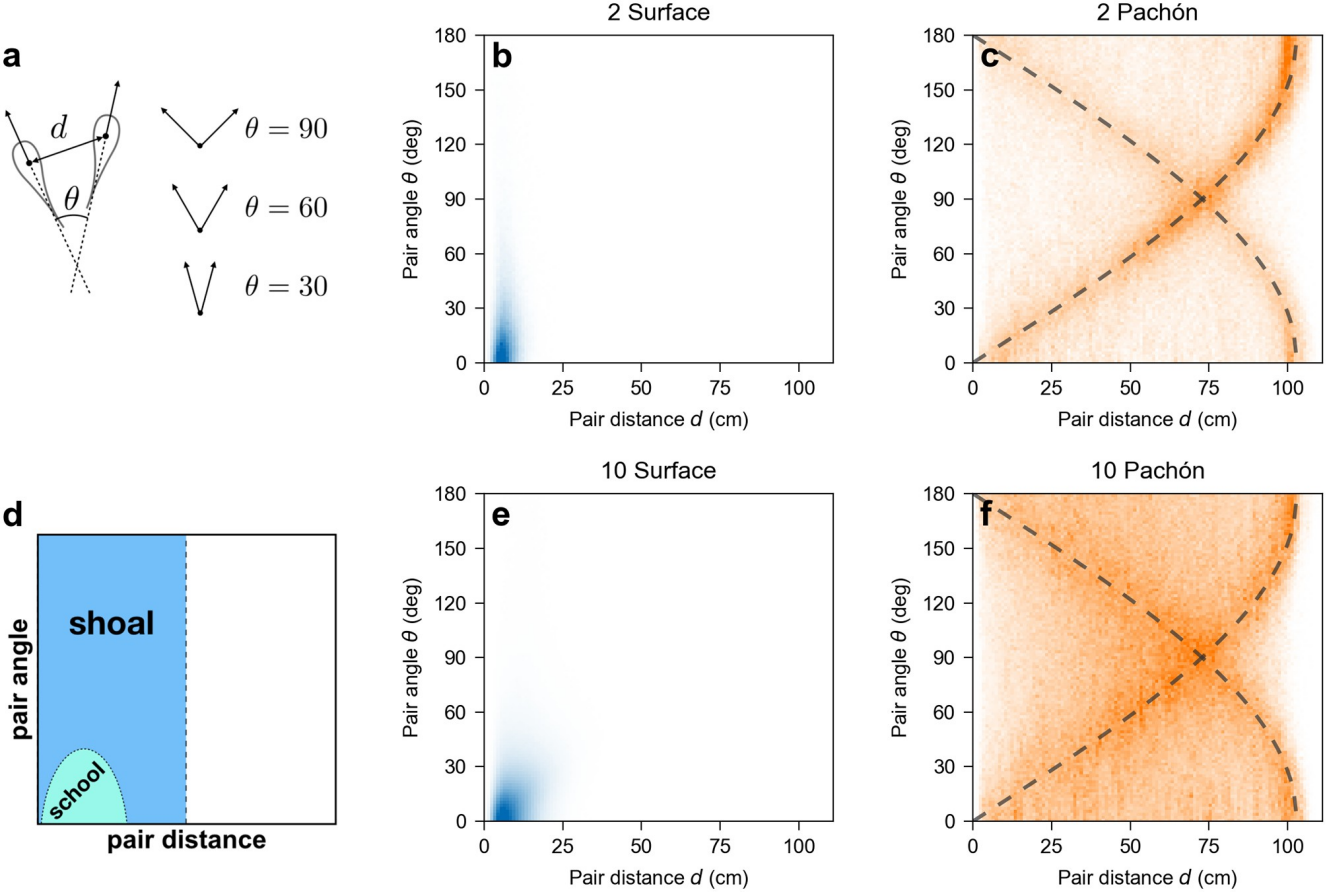
(a) Definition of the pair distance *d* and pair angle *θ* between two fish. (b, c, e, f) Joint probability density of pair distance and pair angle for all pairs of fish. (d) Qualitative expectations for schooling and shoaling fish. For a school, short distances and small relative angles yield a peak in the bottom left corner. For a shoal, short distances and a wide range of angles yield a vertical band on the left side. For a group that does neither, we expect the distribution to spread over the entire available range of distances and angles. (b, e) Groups of surface fish exhibit close proximity and tight angles indicative of strong schooling. (c, f) Groups of Pachón cavefish exhibit neither schooling nor shoaling. The dashed lines show the expectation for fish following the wall. Comparable probability densities are shown for all group sizes and populations (including Tinaja and Molino) in S1 Fig.

When presented with a moving school of artificial fish, surface fish tend to follow it whereas cavefish and vision-deprived surface fish do not [7]. However, little is known about the structure of surface fish schools or the nature of spatial correlations in non-schooling groups of cavefish or vision-deprived surface fish. If the lack of schooling in cavefish is mainly caused by their lack of vision, we expect groups of cavefish and groups of vision-deprived surface fish to exhibit similar spatial arrangements. To address this, we analyze the distance between fish and the angle between their headings (Fig 5).

In surface fish groups, the joint probability distribution of inter-fish distance and angle has a strong peak at short distance and small angle, suggesting that surface fish show strong preference for both proximity and alignment, indicating genuine schooling rather than mere shoaling (Fig 5b and 5e). In groups of two, the distance between the fish is less than 15 cm (3 average body lengths) 96.1% of the time and the angle between their headings is less than 45° 85.5% of the time. In groups of ten, the distance between any two fish is less than 15 cm 75.5% of the time and the angle between their headings is less than 45° 81.5% of the time. Thus, under the light, surface fish in groups spend the majority of their time in close proximity and aligned relative to one another, exhibiting strong schooling behavior.

Unlike surface fish, the joint probability distribution of inter-fish distance and angle in Pachón cavefish reveals neither a preference for proximity nor for alignment (Fig 5c and 5f). The most striking pattern exhibited is a pair of arches due to each fish’s tendency to follow the walls. For fish swimming exactly parallel to the wall at a constant distance *d*_*w*_ from it, we expect $d=\left(R-d_{w}\right)\sqrt{2\pm 2\;\text{cos}\;\theta }$ where *d* is the inter-fish (pair) distance, *θ* is the inter-fish (pair) angle, and *R* is the radius of the tank. Using *R* = 55.5 cm and *d*_*w*_ = 4 cm yields the dashed curves in Fig 5c and 5f. Interestingly, this pattern subsides slightly in larger groups, suggesting that cavefish do not swim along the walls as much when in a larger group. We observed a similar lack of schooling in both Tinaja and Molino cavefish (S1 Fig), suggesting that independently evolved cavefish populations have evolved a loss of schooling behavior.

**Fig 6:**
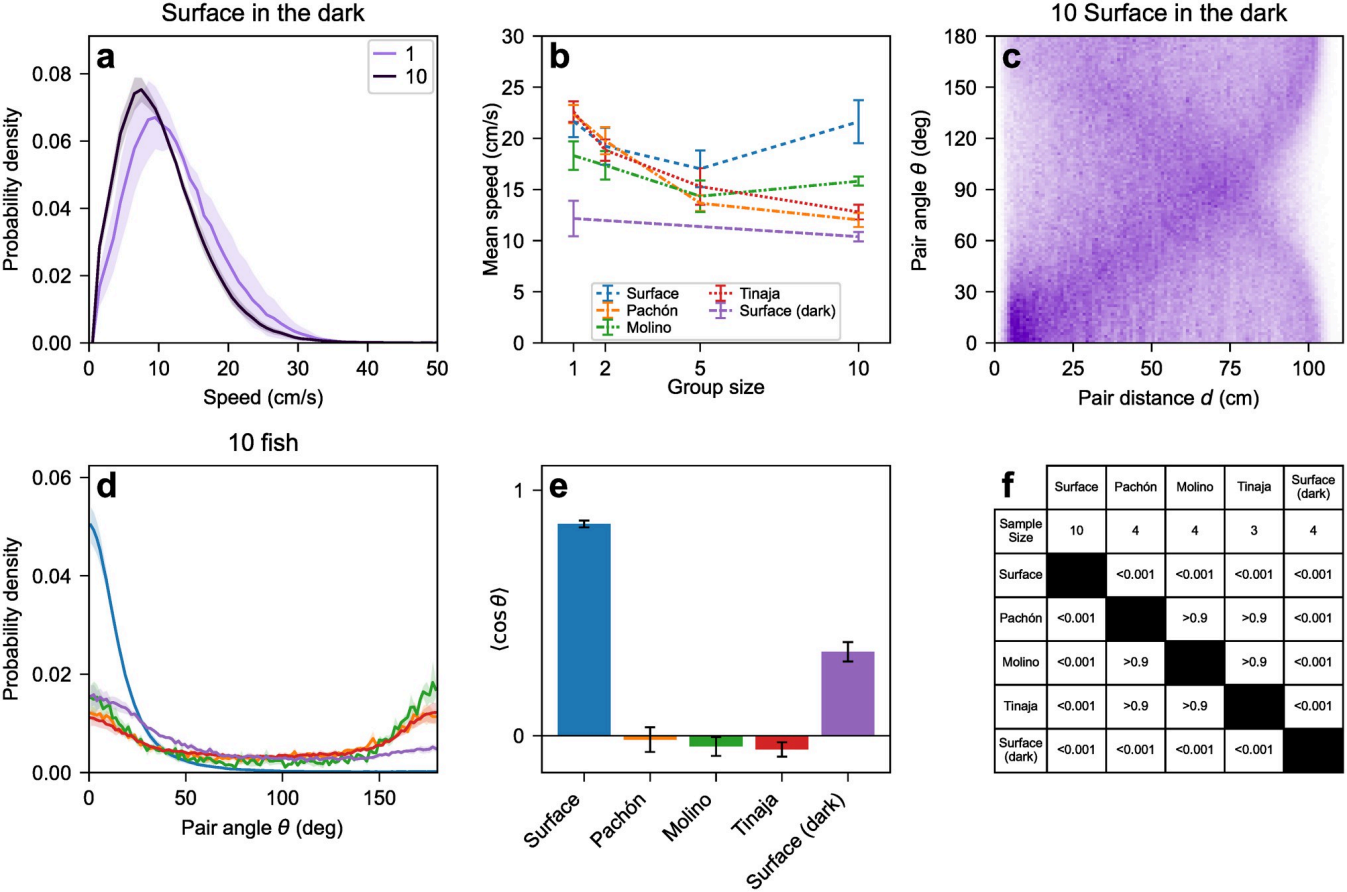
(a) Speed distributions of surface fish in the dark in groups of 1 and 10. The halo around each curve corresponds to one standard error on either side of the mean. (b) Mean speed vs group size for all populations in the light and surface fish in the dark. Surface fish swim slower in the dark than in the light, but their mean speed in the dark shows little change as a function of group size. Linear regression slope *m*_SuDark_ = −0.2 ± 0.2 (*N* = 7, *p* = 0.303). See Fig 3 for the slopes of surface fish in the light, Pachón, Molino, and Tinaja, plotted here again for comparison. (c) The probability density of relative distance and angle exhibits both a spread over the entire tank and a low peak at small distance and angle, indicating locally coordinated swimming but no fully formed school. (d) Probability density of the pair angle *θ* when the two fish are near each other (within 10 cm). The halo around each curve corresponds to one standard error on either side of the mean. (e) Average of cos *θ*, which measures whether pairs of fish tend to move in the same direction (〈cos *θ*〉 > 0) or in opposite directions (〈cos *θ*〉 < 0). (f) Statistics of 〈cos *θ*〉. Surface fish in the dark are significantly less likely to be aligned than surface fish in the light, but significantly more likely to be aligned than any cave population. *N*_SuLight_ = 10, *N*_Pa_ = 4, *N*_Mo_ = 4, *N*_Ti_ = 3, *N*_SuDark_ = 4. One-way ANOVA: *p* < 0.001. Tukey HSD adjusted p-values: *p*_Su-SuDark_ < 0.001, *p*_Su-Pa_ < 0.001, *p*_Su-Mo_ < 0.001, *p*_SuDark-Pa_ < 0.001, *p*_SuDark-Mo_ < 0.001, *p*_SuDark-Ti_ < 0.001, *p*_Pa-Mo_ > 0.9, *p*_Pa-Ti_ > 0.9, *p*_Mo-Ti_ > 0.9.

To find out whether the loss of schooling in cavefish can be explained by their lack of vision, we examined whether cavefish behave similarly to surface fish in the dark. Surface fish in the dark were active (speed > 1 cm/s) 98% of the time, similar to cavefish, but unlike surface fish in the light, which occasionally stop moving when in smaller groups (up to 5 fish, Fig 2). Conversely, the mean swimming speed of active surface fish in the dark is largely independent of the number of fish, similar to surface fish in the light but unlike Pachón and Tinaja cavefish, which swim slower in larger groups (Figs 3, 6a and 6b). Finally, the probability distribution of inter-fish distance and angle of surface fish in the dark exhibits both schooling and non-schooling features (Fig 6c). Similar to the cavefish distribution (Fig 5f), it spreads over the entire available range of distances and angles, indicating there is no long-lived, compact school like the ones observed in surface fish in the light. On the other hand, the distribution does exhibit a peak at short distance and small angle (bottom left corner of Fig 6c) suggesting that vision-deprived surface fish still have a preference for proximity and alignment, even though they no longer form a school. This is confirmed by the distribution of the angle between the headings of two fish located less than 10 cm apart (Fig 6d), which is biased toward acute angles (*θ* < 90°), and the fact that the average cosine of that angle is positive (Fig 6e), indicating alignment is more likely than anti-alignment at close range. Consistent with this interpretation, direct inspection of the video data shows pairs of surface fish in the dark swimming together for brief periods of time but drifting apart before more fish can join this “proto-school”. This is in contrast to groups of 10 cavefish, which show no preference for alignment, even at close range: the distribution of the angle between the headings of two fish located less than 10 cm apart is roughly symmetric around *θ* = 90°, and the average of the cosine of that angle is close to zero, indicating no preference for alignment vs anti-alignment (Fig 6e). Overall, those findings suggest that loss of vision alone is not sufficient to explain loss of schooling in cavefish.

In mathematical models of collective motion, inter-individual interactions favoring proximity and alignment compete with noise (random, unprompted course changes). Strong interactions result in long-range orientational order, i.e., a school. When the interactions are weaker than the noise, one still expects local order in the form of an increased probability for nearby fish to align and/or stay together. This is precisely what we observe in surface fish in the dark. What is more, decreasing the range of the interaction can be sufficient to prevent system-wide alignment [52]. Therefore, our interpretation of the data from surface fish in the dark is that the fish do attempt to school, however the loss of vision reduces their range of perception and prevents them from schooling effectively, consistent with a recent report from another group [53].

### Avoidance behaviors may lead cavefish away from walls

**Fig 7:**
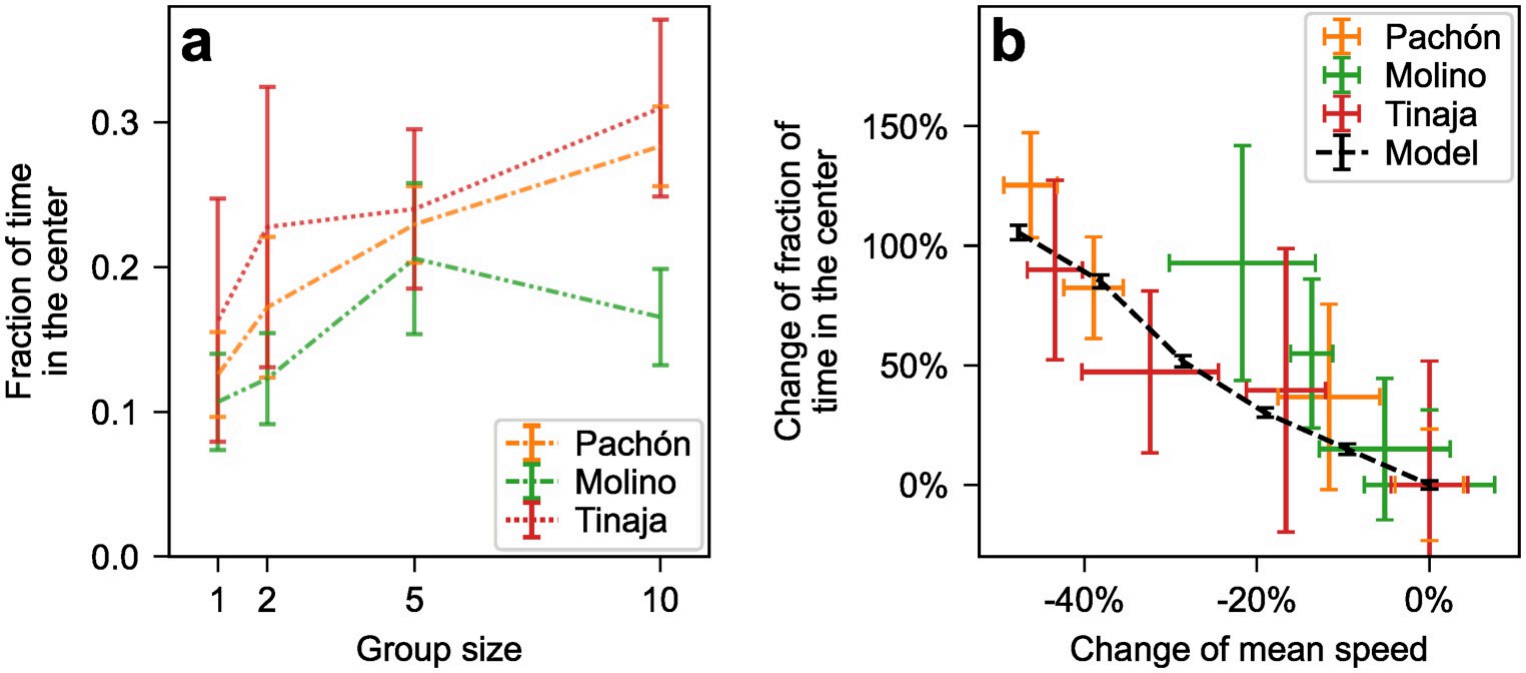
(a) Fraction of time spent in the inner half of the tank (within 39.2 cm of its center, which represents half of its area) as a function of group size in cave populations. (b) Percentage increase of the fraction in the center as a function of the percentage increase of the mean swimming speed in cave populations. Each colored cross represents the average of a specific population and group size. Increase percentages are computed using the isolated-fish mean as reference. This is done independently for each population. As a result, all three isolated-fish crosses (one for each population) are located at (0%, 0%). The black dashed curve shows the results of the computational model.

Wall-following may serve an exploratory function in cave environments [44, 47]. If the walls get overcrowded, however, there may be a benefit to exploring away from them. The qualitative reduction of the wall-following arch pattern in larger groups (Fig 5c and 5f) suggests cavefish do not follow the tank’s wall as much when more fish are present in the tank. To investigate this change, we measure the fraction of the time the fish spend in the center of the tank and use data-driven computer simulations to explore possible explanations for this change in behavior. As expected, the fraction of the time cavefish spend in the inner half of the tank (thereafter *time in the center*) trends upwards with increased group size (Fig 7a).

The tendency to spend more time near confining walls is ubiquitous among motile organisms, even at the micro scale (e.g., *E. coli* bacteria, sperm cells [54, 55]). Studies of abstract self-propelled particle models show that the phenomenon does not require any explicit attraction to the wall, only persistent motion (as opposed to Brownian motion, which constantly changes direction) [56, 57]. This most generic form of the phenomenon is controlled by the persistence length of the motion, i.e., the typical distance traveled in a consistent direction before reorienting. This persistence length in turn is proportional to the self-propulsion speed and to the reorientation time (typical time it takes to change direction). Therefore, we can expect the decrease of the mean swimming speed with increased group size in cavefish (Fig 3c) to explain at least part of the corresponding increase in time spent in the center. Additionally, we can expect behavioral changes that decrease the reorientation time by increasing the frequency or the amplitude of turns to also increase the time spent in the center.

#### Role of the swimming speed

To test the effect of the swimming speed, we first plot the percentage increase of the fraction in the center (compared to isolated fish) as a function of the percentage increase of the mean swimming speed (compared to isolated fish) for the three cavefish populations (Fig 7b). The mean speed decreases by up to 45%, observed in groups of 10 Pachón fish, which also spend 125% more time (over twice as long) in the center compared with isolated Pachón fish.

To further test whether decreased swimming speeds can cause fish to spend more time in the center of the tank, we perform simulations of a simplified model of *A. mexicanus* swimming (see Methods). The simulated fish are propelled by a constant self-propulsion force whose orientation undergoes a random walk (see, e.g., [57]). The diffusion constant of the random walk is extracted from the reorientation time, which is measured from the experimental orientational autocorrelation function of isolated fish and is largely consistent across cave populations. The starting value for the magnitude of the self-propulsion force is the mean swimming speed of isolated fish, averaged over the three cavefish populations. We then vary the self-propulsion speed from simulated trial to simulated trial to cover the range of percentage change seen in experiments (Fig 7b). The interaction with the walls consists of a repulsion force that keeps the fish about half a body length (≈ 2.5 cm) away, and a torque that aligns the fish with the wall when it is moving toward the wall so as to mimic the density peak and wall-alignment effect seen in Fig 1. The strength and the range of the aligning torque are chosen to match the fraction in the center measured in isolated fish (Fig 7a). Since there is no explicit inter-fish interaction and we only vary the swimming speed, the model isolates the effect of the mean swimming speed. In particular, any group size effect that would not act through the mean swimming speed is ignored. Despite this important simplification, the model makes a remarkably accurate prediction for the percentage change of the fraction in the center as a function of the percentage change of the mean swimming speed (Fig 7b, dashed curve). This in turn suggests that the mean swimming speed is indeed a key variable to explain the increase of the time spent in the center as a function of group size in cavefish.

#### Role of the turning speed

**Fig 8:**
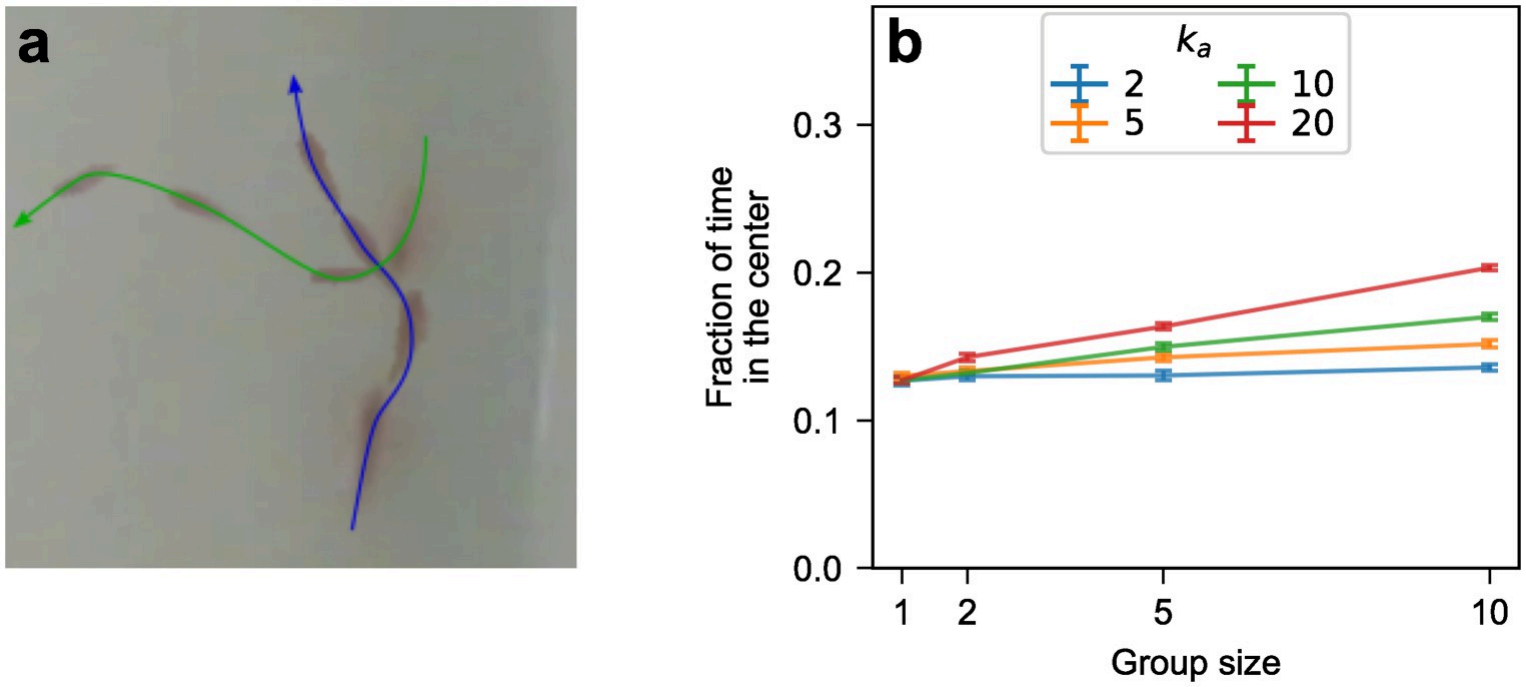
(a) Example of an encounter between two Pachón fish leading to a major change of direction. (b) Fraction of the time spent in the inner half of the tank (within 39.2 cm of its center, which represents half of its area) as a function of group size in simulations including evasive maneuvers. The evasive-maneuver interaction makes fish turn away from each other when they come within half a body length (2.5 cm) of each other. If the strength *k*_*a*_ of the interaction is small, the fish may move out of range of each other before the turn is made. Conversely, if *k*_*a*_ is very large, each fish makes an almost-instantaneous turn to face directly away from the other fish.

When two cavefish get very close to each other, we often observe them react with an evasive maneuver in the form of a sudden wide turn (Fig 8a). This type of close encounter is particularly common at the wall, as fish following the wall in opposite directions are more likely to experience a close encounter in the near future. Evasive maneuvers near the wall are particularly likely to send one or both fish toward the center, therefore impacting the average fraction of time spent in the center.

While a detailed quantification of those evasive maneuvers and their impact on the fraction in the center is beyond the scope of this paper, we can use our computational model to determine whether such maneuvers can in principle increase the time spent in the center. To this end, we supplement our model with an inter-fish interaction that mimics evasive maneuvers: when two fish get within interaction range (half a body length, 2.5 cm, corresponding to close encounters only), they start turning until they either face away from the other fish or are no longer in-range. By changing the strength *k*_*a*_ of the interaction (related to the speed at which the fish turn away from each other, which affects the amplitude of the full evasive turn), we can increase the fraction in the center in groups of 10 fish by up to 60% compared to isolated fish. However, this is only about half of the percentage increase observed in Pachón cavefish, and further increasing *k*_*a*_ does not increase the fraction in the center any further (Fig 8b).

Together, these results suggest that evasive maneuvers do play a role in increasing the time the fish spend exploring the center of the tank, however it is probably a smaller role than that of the swimming speed, and future work is required to better quantify and model those evasive maneuvers.

## Discussion

In this paper, we systematically investigated the collective behavior of *A. mexicanus* individuals drawn from three populations of independently-evolved cavefish and compared them to their extant surface fish relatives, first in the light, then in the dark to simulate sight loss. To generate semi-realistic conditions and allow for the statistical analysis of the spatial structure of groups of fish, we let the fish interact freely in a circular tank that is much larger than those used in previous studies of locomotion and social behavior. We then applied automated tracking [58, 59] to quantify locomotion and interactions between individuals. We identified a series of collective behavioral differences between surface and cave populations that are common to the three cave populations, cannot be explained solely by their common lack of sight, and impact the way they navigate their environment, thus revealing evolutionary convergence on shared mechanisms of collective behavior.

Vision directly influences collective behavior in a range of fish species including mackerel [60], tuna [61], and striped jack [62], among others. Many fish species that show robust collective behavior under normal conditions do not exhibit a complete loss of collective behavior when vision is impeded (via reduced light intensity or a physical barrier covering the eyes), but do exhibit pronounced differences in group structure, such as in saithe [63] and sticklebacks [64]. Therefore, the first point we clarified is the relationship between the loss of sight and the loss of schooling in cavefish. An earlier study of schooling in surface and cave fish suggested that loss of vision plays a large role in the loss of schooling in cavefish, but is probably not the only cause, with additional vision-independent mechanisms also at play [7]. A key piece of evidence for the existence of such vision-independent causes is the absence of schooling in some surface-cave hybrids with seemingly intact visual systems. Here we provide a new, independent piece of evidence that the lack of schooling in cavefish is a behavioral change that cannot be explained solely by the lack of visual input. By going beyond the schooling/non-schooling dichotomy and quantifying the dynamic structure of groups of *A. mexicanus*, we are able to show that the absence of schooling seen in cavefish and the one seen in vision-deprived surface fish are of very different natures: the latter show a preference for proximity and alignment with each other at close distances, suggesting they attempt to school but are unable to, whereas the former exhibit very little preference for proximity or alignment. It follows that the loss of schooling in cavefish is not just a matter of lacking the necessary visual input.

Our quantification of displacement patterns in *A. mexicanus* also reveals that cavefish may actively avoid each other in at least two ways. First, cavefish swim slower when more fish are present in the tank, which mechanically decreases the frequency of their encounters with other fish. Importantly, this is not an instantaneous slowdown based on the current number of nearby fish (a behavior which actually promotes aggregation rather than avoidance [65]). Rather, cavefish seem to respond to the total number of fish in the tank. It is unclear whether that is achieved by some form of remote sensing (e.g. overall level of vibration in the tank), counting the number of recent encounters, or other means. Second, close encounters with other fish often lead to exaggerated turns whose amplitudes appear more consistent with evasive behavior than simple collision avoidance. Regardless of the mechanism(s), those behaviors support the idea that cavefish, unlike vision-deprived surface fish, make no attempt to school.

Interestingly, both avoidance mechanisms (slowdown and evasive turns) promote exploration away from the walls in cavefish. We therefore suggest that they constitute a form of collective response by which groups of cavefish adjust their exploration strategy to the crowdedness of their environment. Depending on the spatial distribution of resources in cave environments, this could provide a fitness advantage that may have contributed to the evolution of loss of schooling in cavefish. Future ecological data on the shape of cave environments and the spatial distribution of cavefish-relevant resources in them would be of great interest and could be fed into our computer simulations to assess the potential fitness benefit of various exploration strategies.

Another goal of our fine quantification of collective swimming patterns in *A. mexicanus* was the identification of potential differences between cave populations. Some traits, like the loss of any preference for alignment with neighbors, were consistent across the three cave populations we studied (Pachón, Tinaja, and Molino). Others, like the avoidance mechanisms promoting exploration away from the walls, exist in very similar forms in Pachón and Tinaja but are absent in Molino fish. Together, this suggests that loss of schooling has evolved independently in different cavefish populations.

The last goal of our study was to make steps towards a mechanistic model of collective swimming in *A. mexicanus*. On the one hand, our results suggest that *A. mexicanus* may be less amenable to highly accurate modeling than some other species, largely because the presence of other fish modifies many aspects of the swimming dynamics, from the speed distribution (including the propensity to swim at all) to the nature of the turns (including surface fish shifting from occasional sharp turns to frequent gradual ones). On the other hand, the mathematical modeling of schooling behaviors is a very active field of research, one in which recent advances may apply to *A. mexicanus* and one in which *A. mexicanus* could push new paradigms. Either way, our analysis of the role of the crowd-induced slowdown and evasive turns in cavefish on their exploration of the tank shows that even simple models that gloss over many of those subtleties can provide valuable insight.

Beyond those specific points, our work illustrates the benefits of generating highly quantitative behavioral phenotypes in evolutionarily tractable model organisms. The ability to quantify diverse aspects of locomotion behavior including velocity, wall proximity, angular velocity, and spatial relationships between fish represent an advance over previous analyses that quantified simple proximity to neighbors or exclusively examined locomotor activity [7, 43]. Our assay and analysis pipeline can be readily adapted to study other behaviors with a kinematic signature, e.g., aggression, sleep, stress and chemosensory behaviors [37, 48, 66, 67]. Fine quantification of those behaviors in surface-cave hybrids should yield new insight into their genetic basis. The recent development of a brain atlas and the mapping of the neuromorphology and neuronal activity in the same four populations discussed in this paper also offers interesting prospects, particularly if we can connect behavioral differences between cave populations to morphological and neuronal differences [68]. Together, this illustrates the potential of *A. mexicanus* as a model organism for the study of behavioral evolution from genetics, to morphology, to neuronal activity, to behavior.

## Materials and methods

### Experimental procedure

#### Husbandry

Animal husbandry was carried out as previously described [69, 70]. All protocols were approved by the IACUC of Florida Atlantic University. Fish were housed in groups in Florida Atlantic University core facilities at a water temperature of 23 ± 1°C in 37.85 L or 75.71 L tanks on a 14:10 hour light-dark cycle. Light intensity was maintained between 24 and 40 lux. Experimental fish were bred in the lab from descendants of either cavefish originally collected from the Pachón, Molino, and Tinaja caves or from two different surface populations originating from Texas or Mexico.

#### Behavioral experiments

All experimental fish were adults housed in groups of 5–10 fish for 10 gallon tanks and 10–25 fish for 20 gallon tanks. Tanks and groups were chosen to avoid repeatedly assaying the same groups of fish and a minimum of two weeks was allowed before assaying fish from a previously used tank. Fish to be assayed were carried to a designated behavior room in a 2.5 L carrier tank and were then gently netted into the experimental arena and allowed to acclimate for 10 minutes.

Experiments were conducted in a round tank (111 cm diameter x 66 cm height) filled to a depth of 10 cm with water taken directly from the tank system. A video camera (Genius WideCam F100, Dongguan Gaoying Computer Products Co., Guangdong, China) was affixed to a custom-built PVC stand that allowed recording from above the center of the tank. Lighting was provided via four white 75-watt equivalent halogen light bulbs (Philips A19 Long Life Light Bulb, Amsterdam, Netherlands) mounted in clamp lights with 5.5 in shades (HDX, The Home Depot, Georgia, United States) to diffuse light. For experiments conducted in the dark, videos were collected by removing the infrared filter from within the camera, and whitelight bulbs were replaced with 940 nm Infrared bulbs (Spy Camera Specialists, Inc., New York, United States) and supplemented with four additional infrared lamps (IR30 WideAngle IR Illuminator, CMVision, Texas, United States). Videos were collected at 30 fps using OBS Studio (Open Broadcaster Software).

### Data collection

Data was first collected in videos that begin recording when fish are released in the tank. The first 10 minutes of video are treated as acclimation time that is later discarded. Analysis focuses on the following 20 minutes. Table 1 summarizes the number of trials and the number of post-acclimation minutes collected for each group size and fish type. Trials were generally run for more than 30 minutes total. However, we have also included a small subset of videos (6 surface and 3 Pachón) despite only having 10 minutes post-acclimation.

**Table 1:** Number of trials collected for each population and group size.

Group Size	Surface (light)	Pachón	Tinaja	Molino	Surface (dark)
1	20	20	4	4	3
2	10	9	4	4	-
5	12	10	4	4	-
10	10	4	4	3	4

### Tracking

The positions and orientations of the fish were extracted from the videos using the custom python tracking library trilab-tracker [71]. It uses adaptive thresholding to locate darker regions with an area and aspect ratio consistent with a fish, and frame-to-frame motion interpolation to maintain the identities of the fish. Fish identities are lost in case of overlap—when two or more fish are detected as a single object.

The circular edge of the tank is selected manually and used to convert pixels to centimeters using the tank’s physical diameter (111 cm).

### Uncertainty due to swimming depth

Although we keep the water depth in the tank small (10 cm), fish can still move up or down by a few centimeters. Given the depth of the water, the height of our camera, and the assumption that fish are generally swimming at half depth, radial positions may be off by as much as $\Delta \equiv \Delta \left(r\right)=\frac{r}{14.2}$. At the wall, this is as much as 3.9 cm, whereas in the center it is zero.

### Kinematics

Time derivatives are computed using a standard second order central finite difference scheme. For example:

$$\begin{array}{cc} v_{x,i} & =\frac{x_{i+1}-x_{i-1}}{2\Delta t} \end{array}$$ (1)

where *x*_*i*_ is the *x* coordinate of the position in frame *i*, *v*_*x*,*i*_ is the x coordinate of the velocity in frame *i*, and Δ*t* = 0.033*s* is the duration of each frame (one over the frame rate).

To compute the angular velocity, the angle difference is first brought back into the (−*π*, *π*] interval:

$$\begin{array}{c} \omega_{i}=\frac{[\left(\theta_{i+i}-\theta_{i-i}+\pi \right)\;\text{mod}\;\left(2\pi \right)]-\pi }{2\Delta t} \end{array}$$ (2)

where *θ*_*i*_ is the angle the fish makes with the *x*-axis in frame *i*, *ω*_*i*_ is the angular speed in frame *i*, and mod is the modulo operator.

### Data filtering

Tracking errors occasionally result in unrealistic speeds or angular speeds. To mitigate the effect of such errors on the overall analysis, we exclude frames in which a fish is moving faster than 100 cm/s or turning faster than 30 rad/s. The cut only affects the fish exhibiting the rapid motion, i.e., the frames are still used to analyze the other fish’s motion. We also exclude occlusion events, i.e., frames in which two fish overlap and cannot be distinguished from each other by the tracking software. For both cuts, we also excludes the three frames preceding the event and the three frames following it to avoid contaminating the kinematic quantities with data from the invalid interval. The inactivity cut discussed in section also uses a three-frame buffer on either side of every inactive interval. Furthermore, if more than half of the frames in a trial are inactive, we remove the entire trial, in accordance with Ref. [7].

### Statistical analysis

All statistical tests were done in Python using Scipy [72], Statsmodels [73] (for Tukey tests), and Pingouin [74] (for Games-Howell tests). Shapiro tests were used to assess normality.

### Simulations

#### Model

The simulation model is based on the 2D Active Brownian Particle model, which has been used to study generic wall-accumulation in self-propelled object [56, 57], with additional forces and torques to capture key fish-wall and fish-fish interactions. The equations of motion for the position **r**_*i*_ and the orientation *θ*_*i*_ of the *i*^th^ fish are:

$$\begin{array}{cc} & \frac{dr_{i}}{dt}=v_{0}\;{\hat{e}}_{i}+\mu F_{i}^{\text{w}}\\ & \frac{d\theta_{i}}{dt}=\eta_{i}\left(t\right)+\mu_{\text{r}}[\tau_{i}^{\text{w}}+\sum_{j}\tau_{ij}^{\text{a}}] \end{array}$$

${\hat{e}}_{i}=\left(\text{cos}\;\theta_{i},\text{sin}\;\theta_{i}\right)$ is the unit vector along the direction of the fish. *v*_0_ is the self-propulsion speed. *η*_*i*_(*t*) is a white Gaussian noise with zero mean and variance 〈*η*_*i*_(*t*)*η*_*j*_(*t*′)〉 = 2*D*_r_*δ*(*t* − *t*′) where *D*_r_ is the angular diffusion constant. It captures unprompted changes of direction. *μ* is the mobility (inverse of the drag coefficient). *μ*_r_ is the angular mobility.

The tank’s wall is modeled as a soft elastic repulsion force with strength *k*_w_ and range *r*_w_. It is zero when the fish is further than *r*_w_ away from the wall (*R* − *r*_w_ − |**r**_*i*_| < 0 where *R* is the radius of the tank), and

$$\begin{array}{c} F_{i}^{\text{w}}=k_{\text{w}}(R-r_{\text{w}}-|r_{i}\left|\right)\;{\hat{r}}_{i} \end{array}$$

when the fish is within *r*_w_ of the wall. ${\hat{r}}_{i}=r_{i}/\left|r_{i}\right|$ is the unit vector in the radial direction.

The fish’s tendency to align with the wall is captured by the wall torque

$$\begin{array}{c} \tau_{i}^{\text{w}}=k_{\text{wt}}\left({\hat{e}}_{i}\cdot {\hat{r}}_{i}\right)\left({\hat{e}}_{i}\times {\hat{r}}_{i}\right)(R-r_{\text{wt}}-|r_{i}\left|\right) \end{array}$$

when the fish is within *r*_wt_ of the wall (*R* − *r*_wt_ − |**r**_*i*_| < 0) and moving towards it (${\hat{e}}_{i}\cdot {\hat{r}}_{i}>0$) and zero otherwise. The product of the dot product and the cross product creates a torque that aligns fish parallel to the nearest wall (perpendicular to ${\hat{r}}_{i}$, which is also the unit normal vector of the nearest wall) without favoring either clockwise or counterclockwise wall-following. The last term makes the torque’s strength decrease linearly as the fish moves away from the wall, reaching zero when the distance from the wall is *r*_wt_.

Evasive maneuvers are captured by the avoidance torque ∑_*j*_ *τ*_*ij*_. The sum is over all the other fish, although only those fish within the avoidance range *r*_a_ actually contribute. The torque acting on fish *i* as a result of the presence of fish *j* is

$$\begin{array}{c} \tau_{ij}^{\text{a}}=k_{\text{a}}\left({\hat{e}}_{i}\times {\hat{r}}_{ij}\right)(r_{\text{a}}-|r_{ij}\left|\right) \end{array}$$

if |**r**_*ij*_| < *r*_a_ and zero otherwise. **r**_*ij*_ = **r**_*j*_ − **r**_*i*_ is the position of fish *j* relative to fish *i*. |**r**_*ij*_| is the distance between the two fish. ${\hat{r}}_{ij}=r_{ij}/\left|r_{ij}\right|$ is the unit vector pointing from fish *i* to fish *j*. *k*_a_ is the strength of the avoidance interaction. The effect of $\tau_{ij}^{\text{a}}$ is to turn ${\hat{e}}_{i}$ (the direction of fish *i*) away from ${\hat{r}}_{ij}$ and towards $-{\hat{r}}_{ij}$; in other words, to turn fish *i* away from fish *j*.

#### Parameters

Lengths are given in centimeters and times in seconds. The radius of the tank was set to *R* = 55.5, same as the experimental tank. The swimming speed parameter *v*_0_ was varied between 9 and 21 so as to cover the range of mean speeds seen in experiments.

The angular diffusion constant *D*_r_ was set to 0.2. To pick it, we measured the autocorrelation function of the swimming direction in isolated fish when they are in the central half of the tank, $\langle {\hat{e}}_{i}\left(t\right)\cdot {\hat{e}}_{i}\left(t^{\prime }\right)\rangle$, then fitted its short-term decay (first second) to (1 − *D*_r_.|*t* − *t*′|). The results were similar across the three cave populations, averaging about 0.2.

The strength and range of the wall repulsion were set to *k*_w_ = 100 and *r*_w_ = 2.5, which essentially prevents the fish from coming closer than 2.5 cm from the wall. The strength and range of the wall alignment were set to *k*_wt_ = 0.83 and *r*_wt_ = 5. Since the fish rarely ever come closer than *r*_w_ from the wall, *r*_wt_ needs to be larger than *r*_w_ for the wall alignemnt torque to have an impact. *r*_wt_ = 5 means that this alignment torque only acts close to the wall (within one body length of the wall). The alignment strength was then tuned to match the experimental fraction of time spent in the central half of the tank for isolated fish: the swimming speed was set to *v*_0_ = 21 (isolated fish average speed, averaged over the three cave populations) and *k*_wt_ was tuned until the fish spent 13% of their time in the central half (experimental average for isolated fish, averaged over the three cave populations). We also tested a larger alignment range (*r*_wt_ = 10), together with a smaller alignment strength (*k*_wt_ = 0.2) in order to maintain the same fraction of time spent in the central half. This had very little effect on the results presented in the paper.

The range of the avoidance torque was set to *r*_a_ = 2.5, reflecting the fact that we only observed evasive maneuvers when two fish come very close to each other. The avoidance strength *k*_a_ was varied between 1 and 50 to covers a wide range of avoidance types, from a barely noticeable deflection when *k*_a_ = 1 to a near-instant turn away from the other fish when *k*_a_ = 50.

#### Integration

The equations of motion were integrated using Euler’s method with time step *dt* = 0.005. The initial condition was obtained by picking a random location along the wall, then placing the fish parallel to the wall at a distance *r*_w_ from it. For Fig 7, we averaged 20 simulations for each swimming speed. Each simulation ran for 200, 000 time units. The first 50, 000 time units were discarded before analysis. For Fig 8, each (*N*, *k*_a_) point where *N* is the group size was obtained by running a single simulation for 10, 00, 000/*N* time units and discarding the first 50, 000 time units before analysis.

## Supporting information

S1 Fig Pairwise maps of collective behavior for all fish populations and group sizes larger than 1 (complements Fig 5). (TIF)
