# Supplementary figures and images for: Kinematic analysis of social interactions deconstructs the evolved loss of schooling behavior in cavefish
Source: PLoS One. 2022 Apr 6;17(4):e0265894. doi: 10.1371/journal.pone.0265894 (PMC8985933; doi:10.1371/journal.pone.0265894)

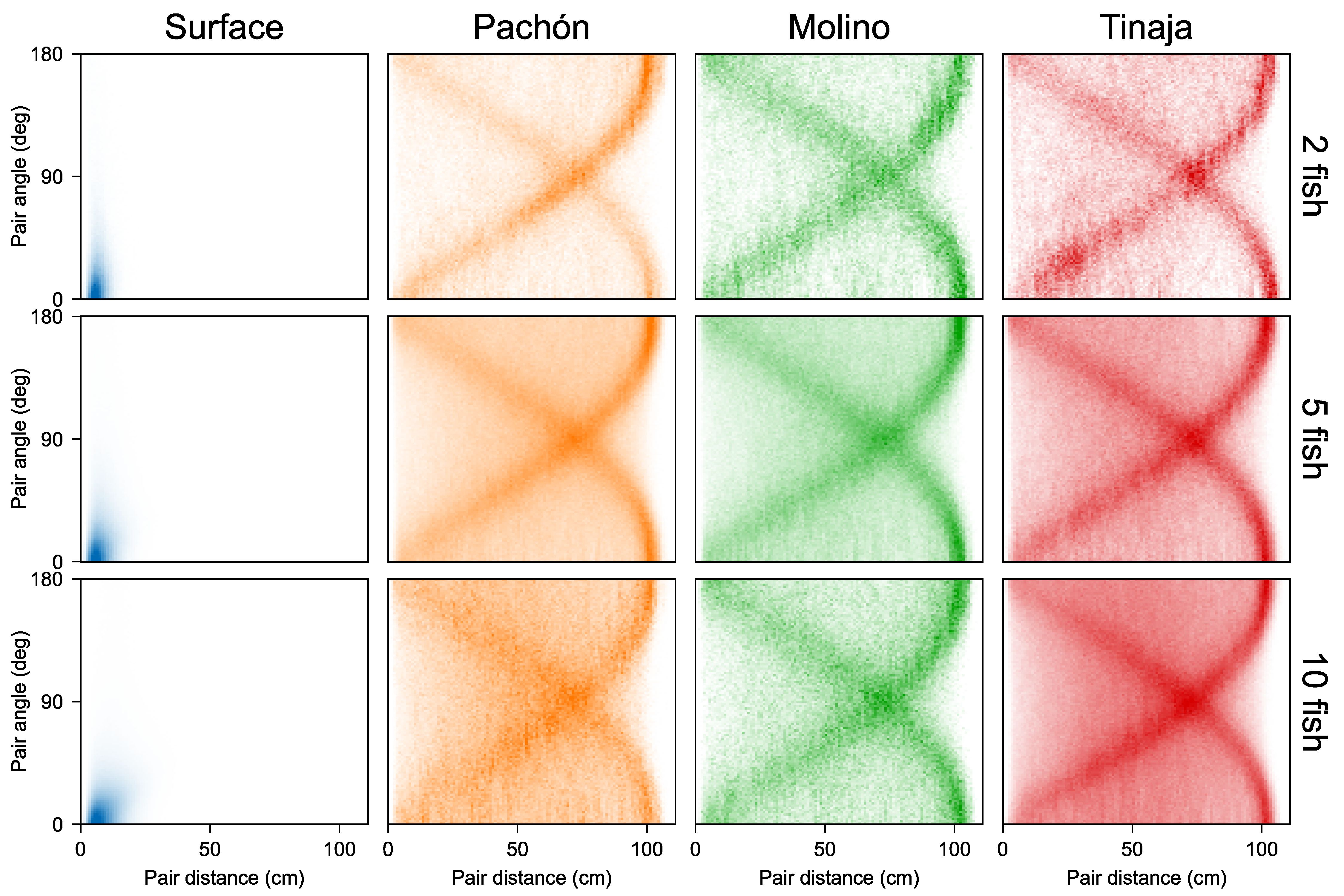

Supplement: S1 Fig — (TIF) [file pone.0265894.s001.tif]
